# Post-menopausal acquired diaphragmatic herniation in the context of endometriosis

**DOI:** 10.1016/j.ijscr.2018.10.024

**Published:** 2018-10-25

**Authors:** Tejas P. Singh, Syed A.A. Rizvi, Casper F. Pretorius

**Affiliations:** a College of Medicine, James Cook University, Townsville, Queensland, Australia; b Surgical Division, Mackay Base Hospital, Mackay, Queensland, Australia; c Surgical Division, The Townsville Hospital, Townsville, Queensland, Australia

**Keywords:** General surgery, Endometriosis, Diaphragmatic herniation

## Abstract

**Introduction:**

Acquired diaphragmatic hernias are most commonly associated with traumatic thoracic injury and rarely heal spontaneously. Conditions that promote peritoneal seeding, such as endometriosis, are associated with spontaneous acquired diaphragmatic hernia formation. Non-traumatic acquired diaphragmatic herniation has previously been described in the context of catamenial pneumothorax, however post-menopausal endometriotic diaphragmatic herniation has not been previously reported.

**Presentation of case:**

A 57 year old post-menopausal female presented with a strangulated ischaemic loop of small bowel herniating through an acquired right sided endometriotic diaphragmatic hernia not previously visualised on imaging. Clamshell thoracolaparotomy was conducted and the necrotic section of small bowel was resected. The diaphragm was repaired and the patient recovered post-operatively without complications.

**Discussion:**

This patient had a complete intestinal malrotation presenting acutely with a small bowel obstruction and herniation through an acquired diaphragmatic rupture. This was possibly related to a diaphragmatic defect caused by endometriosis.

**Conclusion:**

We presented a case of a post-menopausal acquired diaphragmatic herniation secondary to endometriosis; resulting in acute intestinal obstruction and bowel infarction. To our knowledge, such a case has not been previously reported in existing literature.

## Highlights

• Non-traumatic acquired diaphragmatic herniation has previously been described in the context of catamenial pneumothorax.
• Post-menopausal endometriotic diaphragmatic herniation has not been previously reported.
• We presented a case of diaphragmatic herniation secondary to endometriosis; resulting in intestinal obstruction.

## 1 Introduction

Diaphragmatic hernias, generally classified as congenital and acquired, are characterised by the herniation of abdominal structures within the thoracic cavity through a defect of the diaphragm [1]. Congenital diaphragmatic hernias (CDH) are estimated to occur once in every 2000–5000 live births and are the result of inadequate fusion of the septum transversum, pleura and peritoneal folds during the 8th week of embryogenesis [2]. CDH can be further classified as Morgagni and the more common Bochdalek hernias (BH). The latter has a left sided preponderance [3], rarely present in adulthood and are often identified incidentally on imaging [4,5]. BH are caused by incomplete fusion of pleuroperitoneum either posteriorly in the lumbocostal triangles or posterolaterally in the pleuroperitoneal fold. They usually only contain abdominal fat, however larger defects have been shown to contain omentum, bowel, colon, spleen, liver and also the pancreas [4,6]. Majority of BH are detected in utero, or at birth; manifesting as respiratory distress. Furthermore, subsequent gastrointestinal obstruction can present with abdominal pain. Morgagni hernias constitute approximately 3% of CDH and are vulnerable to incarceration. They are characterised by parasternal or retrosternal defects which occur anteromedially in the sternocostal triangle. Morgagni hernias commonly occur on the right side and present symptomatically during adulthood. Surgical repair is the mainstay of management [7,8].

Acquired diaphragmatic hernias (ADH) occur secondary to trauma; blunt, sharp or barotrauma related. Blunt diaphragmatic injury commonly occurs on the left side and has a 40–100% association with concomitant injuries; 90% of these involving thoracic injuries resulting in rib fractures and pneumothorax [9]. Conditions that promote peritoneal seeding, such as endometriosis, are associated with spontaneous ADH formation [10].

## 2 Presentation of case

**Fig. 1:**
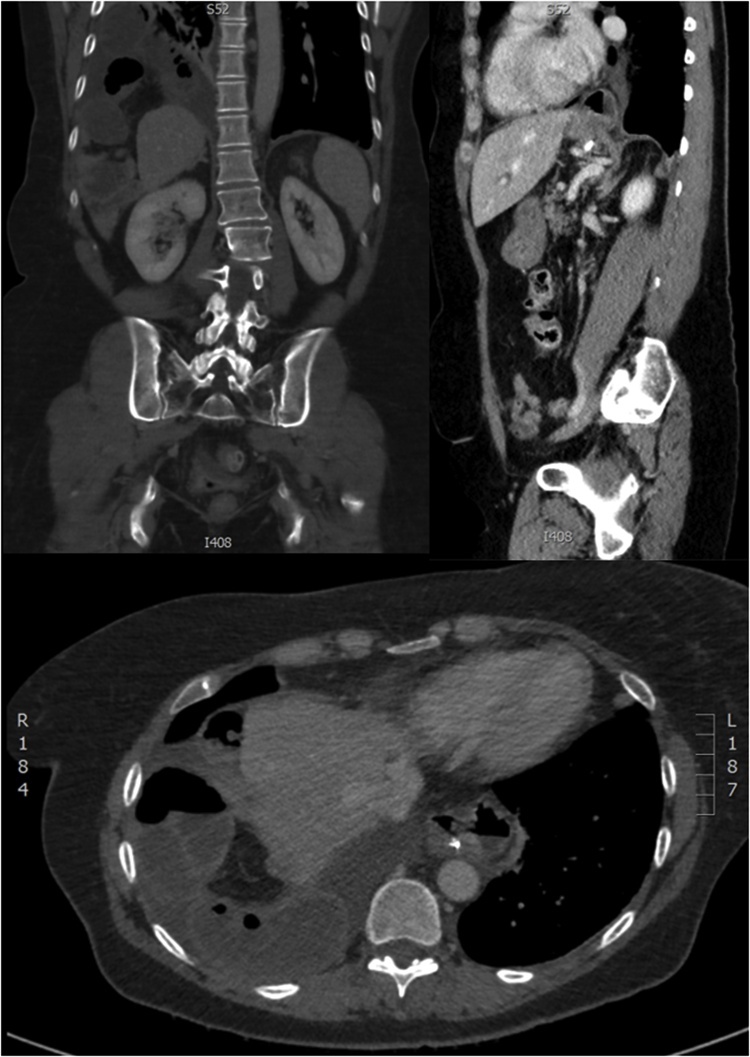
CT images showing defect.

**Fig. 2:**
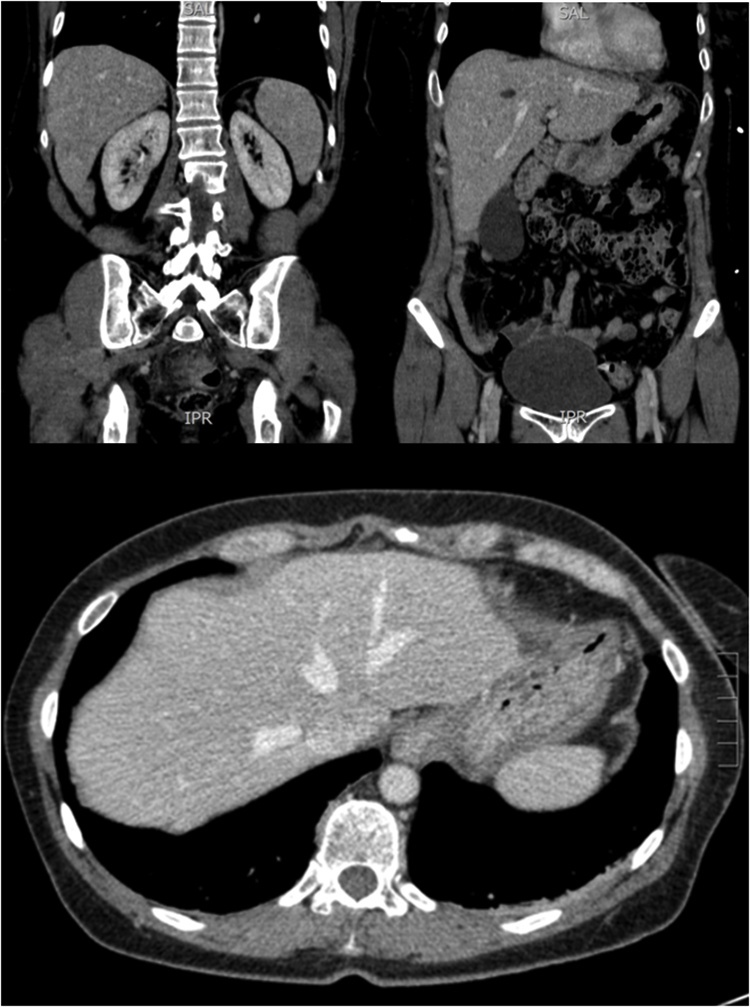
CT images of the same patient 3 years prior to presentation.

A 57 year old post-menopausal female presented with severe sharp hypogastric pain radiating to the flanks and umbilicus. This occurred on the background of endometriosis, congenital bowel malrotation, and previous abdominal surgery. The pain was not alleviated by any positional manoeuvres. Associated symptoms included fever, vomiting and green/bilious sputum. Following admission, the patient recovered significantly with nasogastric decompression and supportive management. A day after admission the pain remitted in the subcostal region; radiating to the flanks and right shoulder. The pain was pleuritic in nature and the patient was subsequently underwent emergency surgery. A strangulated portion of ischemic small bowel herniating through an acquired right sided endometriotic diaphragmatic hernia was identified (Fig. 1). There was no evidence of the defect in previously conducted computed tomography (CT) scans (Fig. 2). Clamshell thoracolaparotomy was conducted and the necrotic section of small bowel was resected. The diaphragm was repaired and the patient progressed well post-operatively without any complications.

## 3 Discussion

Endometriosis is defined as the presence of endometrial-like scar tissue outside the uterus, which induce a chronic inflammatory reaction resulting in scar tissue and adhesions which alter a women’s pelvic anatomy [11]. Endometrial tissue can also manifest intra-peritoneally, intra-thoracically and potentially involve the eye and the brain [12]. It affects approximately 6–10% of the general female population; and has a 25–50% association with infertility [13]. While the exact cause and pathophysiology remains unclear, many theories have been proposed [14]. The retrograde menstruation theory (transtubal migration theory) suggests that seeding endometrial tissue enters the abdominal cavity by way of retrograde menstruation through the fallopian tubes. The coelomic metaplasia theory proposes that metaplastic changes in the germinal epithelium of the ovary can explain occurrences of endometriosis; even in the absence of menstruation [15]. Another widely reported theory suggests that during menstruation, endometrial tissue can implant in the abdominal cavity as a result of endometrial reflux via the fallopian tubes [16].

It has previously been suggested that the clockwise current of intra-abdominal peritoneal fluid can facilitate movement of endometrial tissue to the diaphragm, via the right paracolic gutter; allowing preferential implantation in the right diaphragm [17,18]. Subsequent systemic hormonal changes associated with menstruation can induce implanted endometrial tissue necrosis and eventuate in diaphragmatic perforation. The centrum tendineum has been reported to be the most common area for endometriosis related diaphragmatic defects [17]; as was the case in our patient.

CDH has a significant association with intestinal malrotation in paediatric populations; resulting from intestinal herniation during late foetal development [19]. To our knowledge, no correlation has been found between a non-herniated intestinal malrotation and diaphragmatic defects.

Non-traumatic acquired diaphragmatic herniation has previously been reported in the context of menses-synchronous spontaneous pneumothorax and catamenial pneumothorax. However, to our knowledge, post-menopausal endometriosis related acquired diaphragmatic herniation has not been previously reported in literature. Our patient had a complete intestinal malrotation presenting acutely with a small bowel obstruction and herniation through an acquired diaphragmatic rupture; likely to be secondary to a diaphragmatic defect caused by endometriosis. We believe this case is the first of its type to be reported.

## 4 Conclusion

Non-traumatic acquired diaphragmatic hernias have been described on numerous occasions in the context of catamenial pneumothorax; however post-menopausal endometriosis related diaphragmatic herniation has not been previously reported. We presented the case of a 57 year old post-menopausal female who presented with a non-traumatic acquired diaphragmatic herniation of small bowel causing acute intestinal obstruction.

## Conflicts of interest

There are no conflicts of interest related to this work.

## Funding source

Tejas P Singh holds a Junior Doctor Research Fellowship from the Queensland Government.

## Ethical approval

Ethical approval is not required to publish this case report.

## Consent

Informed consent was obtained from the patient for publication of this case report and accompanying images.

## Author contribution

TPS and AR composed the manuscript; AR & FP contributed to the patients care. All authors have read and approved the final manuscript.

## Registration of research studies

Not applicable.

## Guarantor

TPS and AR

## Provenance and peer review

Not commissioned, externally peer reviewed.
